# A New Perspective on Regulation of Pituitary Plasticity: The Network of SOX2-Positive Cells May Coordinate Responses to Challenge

**DOI:** 10.1210/endocr/bqac089

**Published:** 2022-06-17

**Authors:** Paul R Le Tissier, Joanne F Murray, Patrice Mollard

**Affiliations:** Centre for Discovery Brain Sciences, University of Edinburgh, Edinburgh EH8 9XD, UK; Centre for Discovery Brain Sciences, University of Edinburgh, Edinburgh EH8 9XD, UK; Institute of Functional Genomics, University of Montpellier, CNRS, INSERM, Montpellier F34094, France

**Keywords:** pituitary, plasticity, networks, stem cells

## Abstract

Plasticity of function is required for each of the anterior pituitary endocrine axes to support alterations in the demand for hormone with physiological status and in response to environmental challenge. This plasticity is mediated at the pituitary level by a change in functional cell mass resulting from a combination of alteration in the proportion of responding cells, the amount of hormone secreted from each cell, and the total number of cells within an endocrine cell population. The functional cell mass also depends on its organization into structural and functional networks. The mechanisms underlying alteration in gland output depend on the strength of the stimulus and are axis dependent but in all cases rely on sensing of output of the functional cell mass and its regulation. Here, we present evidence that the size of pituitary cell populations is constrained and suggest this is mediated by a form of quorum sensing. We propose that pituitary cell quorum sensing is mediated by interactions between the networks of endocrine cells and hormone-negative SOX2-positive (SOX2+ve) cells and speculate that the latter act as both a sentinel and actuator of cell number. Evidence for a role of the network of SOX2+ve cells in directly regulating secretion from multiple endocrine cell networks suggests that it also regulates other aspects of the endocrine cell functional mass. A decision-making role of SOX2+ve cells would allow precise coordination of pituitary axes, essential for their appropriate response to physiological status and challenge, as well as prioritization of axis modification.

The anterior pituitary (AP) gland regulates multiple physiological systems with essential roles for maintenance of homeostasis and control of metabolism, growth, and reproduction throughout life. AP hormonal output is not fixed but can alter dramatically with physiological status; for example, the output of growth hormone (GH) transiently increases at puberty to increase statural growth. AP hormone output can also be modified by environmental challenge, such as the increased secretion of adrenocorticotropin (ACTH) in response to stress. These alterations of endocrine output require an appropriate population-level response, with functional modification of each of the hormonal cell types and coordination of homotypic cell behavior. In the short term, changes in output can be explained by acute changes in hypothalamic regulation and peripheral feedback but over extended periods of altered demand functional adaptation at the level of the pituitary is required. This can be mediated by a change in the hormonal output per cell or the number of secreting cells, resulting in modification of the functional mass of a cell population, defined as the maximum output from the population in response to a stimulus.

Appropriate regulation of each of the adaptations that underlie a change in the functional mass of a cell type requires an ability to both sense the secretory capacity of the population and drive its modification. To an extent, hypothalamic regulation and peripheral feedback can fulfill these roles, however, as will be described in this paper, the pituitary response to dysregulation of these inputs indicates an important role for intrapituitary sensing and regulation of population-level function. While this could occur within each endocrine cell population, this would be complicated by the various differentiated hormonal cell types having a common progenitor, as well as a requirement for coordination between the different cell populations to respond appropriately to a physiological challenge. Thus, the capacity to sense, modify, and coordinate the functional mass of each of the populations is likely to include a mechanism that allows intercommunication between different pituitary endocrine cell populations.

Here, we will review the changes in cell function in different pituitary axes that underlie the changes in functional cell mass in response to altered demand, the role of cell networks, and the evidence that the type of response is determined by the strength of demand. We will then present a new perspective, proposing that functional cell mass regulation is mediated by the SOX2+ve cell population that is organized as a structural and functional network. The regulation of multiple axes by this nonhormonal cell type, also described as folliculostellate cells (1), allows prioritization and coordination of the response of the hormone-positive population to challenge. If this proposal is correct, it would suggest a role for cell networks in regulation of cell number, in addition to those previously described for coordination of secretion (2).

## Plasticity of Anterior Pituitary Axes

**Figure 1.**
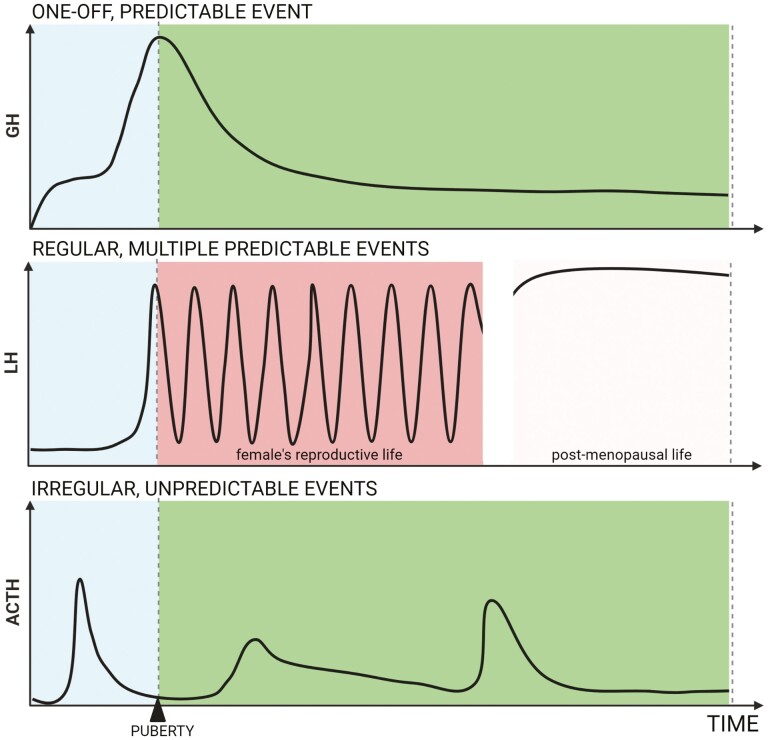
Schematic representation of variation in frequency and predictability of altered demand in different anterior pituitary axes. Top panel: In the growth hormone (GH) axis, the major alteration in demand is a single, predictable event that occurs over a period of years in humans at puberty. Middle panel: In the female, the demand for luteinizing hormone (LH) recurs with a predictable frequency, from puberty until the menopause each reproductive cycle is characterized by a preovulatory LH surge. Bottom panel: In contrast, high levels of demand for adrenocorticotropin (ACTH) occur in response to stress and the onset and duration (acute or chronic) of this challenge is unpredictable and occurs with a varying frequency dependent on environmental cues. Created with BioRender.com.

The frequency, predictability, and duration of alterations in AP hormone output differs between the different hormonal axes (Fig. 1). Dramatically increased GH output is predictable and occurs once at puberty: Before puberty, circulating concentrations of GH are low and after puberty steadily decline with age (3). The increased output of the gonadotrophins, luteinizing hormone (LH) and follicle-stimulating hormone required for ovarian regulation in females, is also predictable but is repeated with each reproductive cycle (4). The principal alteration in requirement for prolactin is in females at lactation (5), which occurs with a varying frequency but since this follows a protracted period of pregnancy, could be considered to be predictable. Each of these challenges varies in duration, from days (gonadotrophins) to years (GH). In contrast, the increased requirement for ACTH and thyrotropin principally occurs in response to environmental challenges, such as stress (6) and low dietary iodine (7), respectively, and are unpredictable, occurring with a varying frequency and duration. These features of pituitary plasticity can also be species dependent, with prolactin and the gonadotrophins varying dramatically with a circannual cycle in seasonal breeding animals (8).

### Mechanisms Underlying Plasticity of Cell Population Function

There are fundamentally 3 population responses that determine hormone output: the proportion of the population of cells that respond to a stimulus, the amount of hormone that each responding cell secretes, and the number of cells in the population. Next, we will describe the evidence for modification to each of these in AP plasticity, not as an exhaustive review but to highlight examples of these mechanisms underlying the response to altered demand.

#### Proportion of responding cells

There is a variable response between pituitary cells of each endocrine axis to stimulation, with heterogeneity of response described in somatotrophs (9), gonadotrophs (10), lactotrophs (11), and corticotrophs (12). While this variability has largely been considered stochastic, recent studies of corticotrophs have described a deterministic mechanism: Repeated stimulation results in consistent responses from each individual cell but responses between cells are distinct (13). Thus heterogeneity in a population of cells may be one mechanism that controls population response to a stimulus, and in the longer term a shift in the proportion of responding cells may alter hormone output. Indeed, a steroid-driven increase in the proportion of somatotroph cells and hormone secretion in response to GH-releasing hormone (GHRH) has been shown in studies of mouse pituitary slices (14). Similarly, an increase in the proportion of lactotroph cells with high calcium activity has been shown in lactating mice compared with virgin animals, which returns to prelactation levels after cessation of demand at weaning (15). Previous studies have shown pituitary cell heterogeneity at the level of secretion of multiple hormones and response to secretagogues (eg, [16-19]) and single-cell transcriptomics has supported the existence of distinct subpopulations that are functionally diverse (20-22). Whether these subpopulations of cells permanently differ in their function or are cells that dynamically transition between several states of activity remains to be determined (see “Future Studies”).

#### Altered secretion from responding cells

Whether or not the proportion of secreting cells changes, over an extended period of altered demand (multiple hours to days, weeks) there must be a change in the amount of hormone secreted per cell in the absence of changes in cell number. This is the case for lactotrophs of lactating mice, in which the dramatically increased calcium activity in cells indicates an increased output of prolactin (15). In male mice somatotrophs respond to GHRH stimulation with a markedly higher GH secretion compared with females, a change that corresponds with a distinct calcium response in individual somatotrophs (14). The mechanisms underlying changes in the sensitivity and quantity of hormone secreted have been best characterized in gonadotroph cells during the ovarian cycle (recently reviewed in [4]). Increased secretion is driven by a heightened sensitivity to gonadotrophin-releasing hormone mediated by an increase in receptor expression and altered intracellular signaling following receptor activation. This leads to an upregulation of LH gene expression and secretory granule release, as well as altered cell morphology, with increased apposition of the secretory surface to the vasculature (4).

#### Changes in the number of cells

Cell number determines the maximal secretion from any of the pituitary hormone cell populations (since an individual cell will have a ceiling of secretory capacity). Thus, it might be expected that a sustained alteration in demand would be associated with an alteration in cell number and that in systems where this change in demand is predictable, this would precede the requirement for altered hormonal output. This has been described for rat lactotrophs, with increased cell number from 15% to 50% of the total pituitary cell mass in pregnancy, which returns to the nulliparous population size within 7 to 21 days of weaning (23). This is not a consistent mechanism even within one pituitary axis, since a dramatic increase in lactotroph cell number has been shown not to occur in lactating mice (15, 24, 25). Similarly, the predictable and dramatic circannual increases in prolactin and the gonadotrophins in seasonal breeding animals is not a result of increased numbers of either lactotrophs or gonadotrophs in sheep or horses (8).

Perhaps counterintuitively, the system by which a change in cell number is most closely correlated with demand is in thyrotrophs, where the alteration in demand is unpredictable and driven by environmental factors. In response to hypothyroidism, the number of thyrotrophs in rats and mice increase dramatically (26, 27) and in rats occurs by both transdifferentiation of somatotrophs (26, 28) as well as cell proliferation (29). A similar mechanism involving somatotroph transdifferentiation has been described in humans with hypothyroidism (30); however, the stimulatory effects of thyroid hormone on GH gene expression (31) means that potential silencing of GH expression in multihormonal cells cannot be distinguished from bona fide transdifferentiation. On return to a euthyroid state, the thyrotroph population rapidly returns to a prechallenged state (26).

The question remains of how these changes can be coordinated to achieve precise regulation of the population: Such coordination would require would require mechanisms that regulate the population response to demand and also monitor output of the whole population.

### The Role of Cell Networks in Anterior Pituitary Plasticity

The mechanisms of altered AP output described earlier consider cell autonomous changes, without coordination of the cell population. However, our groups have shown that pituitary cells are organized as homotypic cell networks (2), which mediate coordination of the response of cells to stimulation (14). In thick pituitary slices from male mice, with preservation of the structural organization of somatotrophs as clusters linked by strings of homotypic cells that form a continuum within the gland, calcium activity in response to GHRH is coordinated in a large proportion of cells, with waves of calcium activity that continue after cessation of stimulation. The role of the structural network is demonstrated by loss of coordinated calcium activity where the somatotroph network has failed to form, as a result of loss of GHRH-driven cell proliferation, or following enzymatic cell dispersion. A similar network-mediated coordination of cell function has been shown for lactotroph cells, where specific cells acting as hubs have been shown to drive coordination in the network through gap-junction mediated coupling (15). In addition to modification of secretory activity, network-mediated coordination of lactotroph gene expression has been described previously (32).

The organization of the various homotypic cell networks are distinctly different (33), suggesting that, in addition to ensuring cell-cell contact, the topological organizations are functionally relevant, influencing cell population hormone output and the plasticity of the endocrine systems. Consistent with this is a reversible change in the organization of somatotrophs in male animals associated with the increased output of GH at puberty (34). In addition, the organization differs between male and female mice and is altered by gonadal steroids (35), with these changes in organization resulting in altered functional coordination and hormone output (14).

Plasticity in network organization associated with altered demand has also been shown in lactotroph cells in mice (15), with increased structural organization in lactation resulting in enhanced coordination of cell activity through a higher number of cells acting as hubs to drive network functional output via increased gap-junctional coupling between the lactotrophs. Remarkably, this altered structural and functional organization of lactotrophs is maintained for months after weaning and results in a further increase in coordination and hormone output in subsequent lactations. Thus, unlike somatotrophs, changes in the lactotroph network are not reversible but retain a functional memory of previous demand and an ability to “learn” by augmenting output on subsequent challenge, consistent with a system in which challenge is likely to be repeated. This may be common to other pituitary axes with cycles of high and low levels of hormonal output.

The role of network organization in coordinating homotypic cell population secretory output and anticipating repeated demand makes them an important feature of the functional mass of each endocrine cell type. However, their role in the regulation of cell number is currently unclear.

### Regulation of Functional Cell Mass Depends on the Strength of Challenge

Lactotroph cell network plasticity is dependent on the strength of the challenge to the system, with a reduction in demand achieved by decreasing the number of suckling pups resulting in a loss of structural and functional changes (15). This dependence of plasticity on the strength of the demand for increased output is consistent with changes seen in other pituitary cell types in mouse models. Increased demand for ACTH in chronic stress has been shown to result in corticotroph cell proliferation in stress paradigms inducing a high level of demand for ACTH (36) but not in response to mild stress (21). Adrenalectomy, which may be considered an extreme challenge to the corticotroph system but is similar to that experienced by patients with Addison disease, also results in proliferation of corticotrophs (37). Similarly, proliferation of gonadotrophs is most abundant following end-organ ablation by gonadectomy (38, 39). Thus, duration of demand, as well as strength and target organ feedback, may determine the mechanisms supporting plasticity: Cell proliferation occurs in response to a prolonged requirement for very high levels of hormonal output, and there may be a threshold of increased cell activity that triggers this.

Again, the mechanism(s) underlying this are unclear, although expression of several of the receptors for the hypothalamic factors that are the main drivers of proliferation are restricted to differentiated hormonal cells (40, 41). This suggests that the hormonal cell type itself must play at least an intermediary role in the decision to proliferate. However, it is less clear whether this may include input from other pituitary cell types (discussed next).

### Anterior Pituitary Cell Mass Is Constrained

Multiple models in rodents with a high level of drive for proliferation of pituitary cells show that differentiated cell number is constrained, despite continued stimulation. In the end-organ ablation models described earlier, an increase in corticotroph or gonadotroph number occurs for only a limited period of time, despite continued lack of target organ steroid (37, 42). This is not due to a lack of steroid sensitivity, as steroid replacement rapidly reduces the cell population to the numbers found in intact animals (37, 38). Other models of increased hypothalamic drive but with intact end-organ function also result in pituitary cell proliferation; however, again the increase in cell number plateaus despite continued stimulation (43, 44). Transgenic overexpression of GHRH, with high levels of ectopic expression within the pituitary (43), results in somatotroph hyperplasia but the increased somatotroph cell number remains static between ages 2 and 6 months, although there is further expansion and adenoma formation in a proportion of animals at age 1 year (44). This constraint on somatotroph hyperplasia is not due to altered GH axis feedback, as combining the transgenic overexpression of GHRH with knockout of the GH receptor does not result in a further expansion of somatotrophs. Similarly, loss of dopamine signaling in lactotrophs through knockout of the dopamine 2 receptor leads to cell hyperplasia that is constrained, with cell numbers increasing in young animals but then remaining stable for a protracted period before eventual adenoma formation (45). Thus, even in the presence of strong trophic stimuli, the population size of pituitary cells is constrained, suggesting an intrapituitary mechanism monitoring cell type number and constraining their proliferation.

While monitoring of population size clearly requires an input from the specific cell type itself, the multiple demands on the pituitary and axis crosstalk suggests a degree of coordination is required. For example, the expansion of thyrotrophs in response to hypothyroidism that also results in a loss of somatotrophs requires the coordinated monitoring of 2 populations. This may occur through interactions between each of the hormonal populations, but the multiple scenarios in which a coordination of cell number modification is required raises the possibility of a contribution from a nonhormonal cell type that is able to make targeted, selective interconnection with most, if not all, networks of differentiated endocrine cells.

## New Perspective: Functional Cell Mass Sensing and Regulation of Pituitary Plasticity

Regulation of each of the AP axes requires population-level coordination to ensure appropriate output from the gland both during homeostasis and in response to challenge. Over extended periods of time, challenge results in an altered set point of output through a change in functional cell mass and each of the mechanisms underlying pituitary plasticity could be modified to achieve this. It is possible that the coordination required for this change in functional cell mass may be through a network sensing its own capacity and integrating hypothalamic and peripheral inputs through intranetwork mechanisms: Modeling of other endocrine systems has shown that this allows a dynamic response to challenge (46). However, the interrelationship of the various endocrine cell networks and the crosstalk required for coordinated output of multiple axes suggests additional mechanisms are required. A second possibility is that external cues that regulate multiple AP endocrine axes are integrated by a nonendocrine network that then selectively signals to specific endocrine networks to modify their functional cell mass. We propose that the second possibility provides an efficient mechanism with increased robustness and optimization of axes’ crosstalk. An excellent candidate for this nonendocrine cell type is the SOX2+ve cell population, which forms an extensive reticular-like network that extends throughout the AP, allowing homotypic SOX2+ve cell communication over a wide range of distances within the pituitary parenchyma (47, 48) and precise bidirectional communication with distinct endocrine cell networks (1, 33).

The SOX2 transcription factor is expressed in the embryonic progenitors of the AP endocrine cells and is essential for normal proliferation and differentiation of these cells in development (49, 50). In adulthood SOX2+ve cells have been identified as a potential pituitary stem cell population (reviewed in [51]), acting as a source of a proportion of newly differentiated endocrine cells, thereby contributing to tissue homeostasis as well as the expansion of specific endocrine cell types in response to end-organ ablation (52, 53). Elegant studies have revealed a further role, with SOX2+ve cell WNT secretion driving proliferation of committed progenitors of all AP endocrine cell types (54). Modeling of tissues with multiple cell types that exchange growth factors, such as the AP, has shown that heterotypic interaction between 2 cell populations can result in stable and resilient regulation of cell populations (55). The SOX2+ve population overlaps extensively with that described as folliculostellate cells (reviewed in [1]), which have previously been described as a source of growth factors regulating multiple endocrine cell populations (48). This is consistent with their network-coordinated activity having a major role in the resulting cell circuits regulating gland function.

### Network of SOX2-Positive Cells as Sensor and Regulator of Pituitary Cell Mass

In common with all tissues, the homeostatic maintenance of AP population size requires a balance of rates of proliferation and apoptosis of each pituitary cell type (56). The postnatal turnover of pituitary cells is illustrated by lineage tracing studies (54) and, as described earlier, the population size can vary in response to demand but is constrained. This suggests that within a gland with limited capacity for expansion, a mechanism must exist that can monitor cell density, that is quorum sensing (57), classically described in bacteria but more recently in postnatal regulation of the immune system (58) and stem cell populations (59). Modeling of these systems has shown that growth factor exchange between multiple cell types can lead to stable, robust cell circuits that resist perturbation (55) but also allows for a modification of cell population size to a new, stable set point, as occurs in pituitary models with a strong hyperplastic drive. A further feature of this modeling is the concept that 1 of the 2 cell types in the heterotypic circuit is present at its “carrying capacity,” that is, the limit of the population size that can be supported by the tissue environment. Over time, however, this carrying capacity can be modified by a change in the environment, such as a change in vascularization, altering pituitary oxygen or nutrient supply (60).

We propose that the SOX-2+ve cell network is at its carrying capacity and that its paracrine interactions regulate the population size of each of the endocrine cell networks. The extensive network organization of the SOX2+ve cell population allows it to precisely deliver local information to distinct endocrine cell types, which are organized as intermingled cell networks, through its intranetwork communication and precisely defined contact zones with distinct endocrine cell types (61, 62). This would predict that the SOX2+ve cell network i) produces signals that regulate the endocrine cell populations; ii) responds to signals from the endocrine cell populations; and iii) responds to external factors to modify the carrying capacity of the SOX2+ve population and allow finely tuned expansion of an endocrine cell population in response to altered demand. The identification of the SOX2+ve cell population as a source of WNT proteins (54), as well as other secreted factors such as interleukin 6 (20) and other paracrine factors regulating AP endocrine cell populations (48), suggests that the first prediction can be met. Thyrotropin receptors are expressed in SOX2+ve cells (63), and analysis of single-cell sequencing data sets reveals an enrichment of GHR in this cell type (Linda Laiho, University of Edinburgh, personal communication December 7, 2021). SOX2+ve cells also express a range of other receptors for potential paracrine factors from other pituitary cell types (48). Thus it is clear that the SOX2+ve cell network has the ability to respond to secreted factors from endocrine cell networks. Finally, the regulation of SOX2+ve cells by a range of peripheral factors, such as glucocorticoids, has been described previously (recently reviewed in [1]), fulfilling the final prediction.

Our proposal that Sox2+ve cells act as sentinels and regulators of the functional mass of pituitary endocrine cells would also suggest that their ablation would have considerable effects on gland function and its ability to respond to challenge. This has been assessed in an elegant study by Roose and colleagues (64) using diphtheria toxin to temporally ablate Sox2+ve cells, with surprisingly little effect either on hormonal cell number or the ability of corticotrophs to respond to the challenge of adrenalectomy. While this may at first appear to counter our suggestion of a role for the Sox2+ve cells in regulation of pituitary functional cell mass, significant ablation in this study was principally achieved in the subpopulation of Sox2+ve cells of the marginal zone of the gland, leaving a large proportion of cells in the parenchyma of the gland unaltered. These parenchymal Sox2+ve cells, intermingled with hormone cell networks, are ideally placed to fulfill the role that we propose as sentinels and actuators of endocrine cell number.

### Network of SOX2-Positive Cells as Sensor and Regulator of Pituitary Cell Secretory Activity

**Figure 2.**
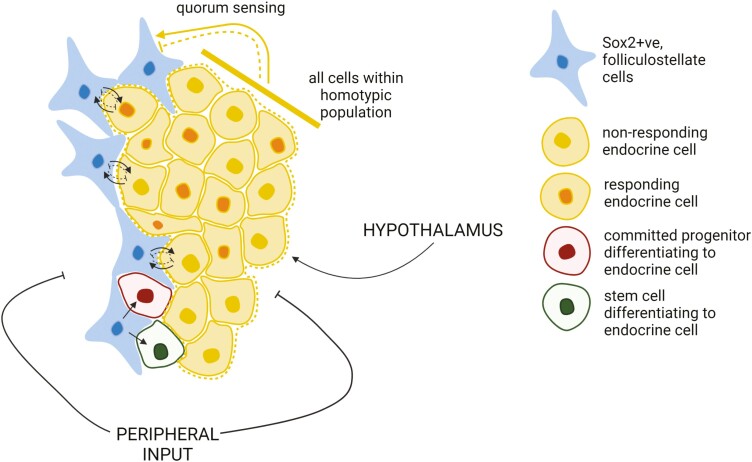
The functional mass of anterior pituitary endocrine populations is regulated by dynamic interactions with SOX2-positive (SOX2+ve) cell network. The population-level response to hypothalamic and peripheral regulation of endocrine cells is dependent on its functional mass, which is determined by the number of cells, the proportion of cells that respond to stimulation, the amount of hormone they are able to secrete, and structural organization that coordinates network function. Each of these is regulated by dynamic interactions with a network of SOX2+ve cells, which may act to sense the quorum of an endocrine cell type thereby actively altering cell number by either acting as a source of additional endocrine cells and/or regulating the generation of additional endocrine cells by differentiation of committed progenitors. This idealized figure represents the interactions of just one of the endocrine cell networks with the SOX2+ve cell network and does not include the interactions with other endocrine networks or the intranetwork signaling that coordinates SOX2+ve cell function. Created with BioRender.com.

Our prediction that the SOX2+ve cell network has an important role as a sensor and regulator of AP endocrine cell population size could also be extended to regulation of the other features that regulate the output of the endocrine cell networks, the proportion of responding cells, and their secretory activity. For example, the altered externalization of annexin 1 in SOX2+ve cells in response to glucocorticoid feedback has been shown to reduce ACTH secretion from corticotrophs in response to corticotrophin-releasing hormone (65, 66). A role in the regulation of the dynamics of hormone secretion would also clearly be supported by their functional network organization that supports coordinated calcium signaling (47, 67). Thus, it is possible that this network of SOX2+ve cells is able to interact with the endocrine cell networks and regulate the multiple, demand-dependent mechanisms, leading to plasticity of hormonal output and functional cell mass (Fig. 2).

### SOX2-Positive Cell Network as Driver and Coordinator of Endocrine Cell Plasticity

If SOX2+ve cells fulfill the roles that we have proposed earlier, then 2 important questions arise: Are SOX2+ve cells the regulators or are they passive facilitators regulated by endocrine cells, and how do SOX2+ve cell regulate several pituitary axes? A hierarchy of cell regulation within the pituitary is suggested by aspects of axes crosstalk. In end-organ ablation studies in rats, it has been shown that thyroidectomy decreases the corticotroph response to stress in adrenalectomized rats (68), while concurrent adrenalectomy and gonadectomy results in a delayed gonadotroph response compared with gonadectomy alone (69, 70). Further analysis of gonadectomized and adrenalectomized animals reveals a hormone-negative cell population that preferentially proliferates to generate corticotrophs rather than gonadotrophs if both organs are ablated (71). As SOX2+ve cells both regulate and act as a source of newly differentiated endocrine cells, it is most likely that they are the cell type prioritizing which endocrine cell population increases in response to multiple challenges and identifies them as regulators rather than passive facilitators. The crosstalk between the adrenal and gonadal axes is well established (72) and there is clear potential for a role of SOX2+ve cell regulation of multiple aspects of gonadotroph and corticotroph function in contributing to this.

The potential role for SOX2+ve cells in crosstalk between pituitary axes emphasizes the importance of the question of how this cell type is able to fulfill multiple roles and regulate multiple cell populations. There may be multiple SOX2+ve cell populations, each with a distinct role in regulating individual endocrine pituitary cell types; however, recent single-cell RNA sequencing (scRNAseq) studies of the pituitary describe the SOX2+ve population either as a single cell type (21, 22, 25) or, at most, 2 distinct populations (20). Thus it is more probable that functional heterogeneity within the population exists with an individual cell having a distinct pattern of gene expression and role that is plastic (1). The network organization of SOX2+ve cells (47) and the interdigitation of this with that of the endocrine cells allows both endocrine cell type–specific regulation and coordination of axes crosstalk over the entire pituitary, as well as minimizing the effects of local functional perturbation. This topographical organization would suggest communication from SOX2+ve cells over a short distance, which may be important to allow functional modification of a specific cell type (see Fig. 2), facilitated by the morphology of SOX2+ve cells that would allow specific actions: Thin processes of SOX2+ve cells with regional concentration of growth factors make bouton-like contacts with hormonal cells (73). The homotypic organization of SOX2+ve cells and relationship with hormonal networks, including precise regional delivery of growth factors, allows adoption of diverse roles by this cell type and exquisitely specific regulation of multiple cell types.

## Future Studies

While aspects of pituitary cell plasticity in response to demand have been described for the different pituitary cell types, it is also clear that for each there are gaps in our knowledge. The consequence of ablation of all Sox2+ve cells (74), including those in the parenchyma, or disruption of the network organization of parenchymal SOX2+ve cells would establish whether our proposal of their central role in gland function is valid. This may require identification of specific markers of parenchymal and marginal zone Sox2+ve cells; however, scRNAseq data sets are being generated that should allow this (eg, [21, 74, 75]). Other gaps in our understanding of pituitary plasticity may be filled by these analyses of pituitary cells using scRNAseq analysis, which includes studies of pituitary responses to physiological challenges (20-22, 25). These will identify functional heterogeneity of pituitary cells but will also allow identification of paracrine interactions through analysis of the distribution of secreted factors and their receptors, as well as intracellular responses (76) and more detailed understanding of the crosstalk between axes in various physiological conditions. A more detailed understanding of how cell-type specific interactions occur with the network of SOX2+ve cells may result from gene expression studies, in particular using newer methodologies that allow spatial as well as cell-type analysis (77). scRNAseq studies have also questioned the dogma of single-hormone cell types and rekindled an interest in the possible role of transdifferentiation; that is, a cell stopping the production of one hormone and starting the secretion of another (eg, a somatotroph switching to a lactotroph). Lineage tracing studies conducted to date have suggested that transdifferentiation is very limited (24, 78, 79) but most have not included physiological challenges in their analyses: Further study of the potential for cell type plasticity is warranted, which may include the type of manipulations used by Odle and colleagues (79) to dramatically alter pituitary function. A role for transdifferentiation in pituitary plasticity would also provide a rationale for the “reserve” of pituitary cells, whereby only a proportion of cells are required to support the hormone secretion required for appropriate physiological function (80, 81).

Further characterization of the intact gland and its function will be required to further understand the fundamental mechanisms underlying altered population-level output from the AP gland. While aspects of this can be addressed by existing approaches, more recent technical developments, in particular in vivo imaging of the pituitary (82), will allow more detailed analyses. The power of these in vivo imaging techniques is their ability to establish how pituitary plasticity occurs in more physiologically relevant contexts; that is, the gland in situ, with intact aspects such as blood flow and in conscious, freely moving mice. Such imaging approaches also allow monitoring of animals over an extended time period, with analysis of changes in response to physiological challenge at a temporal resolution that could not be achieved previously, with animals acting as their own control. The role of the vasculature in pituitary plasticity, indicated by the remodeling that occurs in seasonal breeding animals (60), could also be addressed using these imaging approaches.

Proof of mechanisms underlying functional plasticity will require the generation of mouse models showing the effects of perturbation. To date, most of these have had limited capacity for temporal, tissue, or cell-type specificity. The development of the use of viral transduction (82) may overcome some of these limitations, particularly if CRISPR-Cas9 approaches can be used to modify expression and cell function (83). These have the potential to provide a quick and relatively low-cost strategy to determine the role of specific patterns of gene expression in combination with new in vivo studies and single-cell multiomics. This would allow dissection of the importance of the multitude of gene variants recently unveiled in genome-wide association studies and whether their combination causes pituitary defects related to dysfunction in the crosstalk between the network of SOX2+ve cells and other pituitary cell networks. These studies should include a consideration of the distribution of growth factors within the tissue parenchyma, as well as how this may be altered by blood flow and modification of the extracellular milieu. In addition, a combination of in vivo imaging with CRISPR-Cas9 may allow elucidation of the importance of growth factor interactions between multiple cell populations in prevention of adenoma formation, as models of growth factor interaction suggest that this may lead to the elimination of cells with mutations that would otherwise result in tumors (84).

## Conclusions

Understanding of the mechanisms underlying pituitary plasticity has advanced substantially but is fragmented. While for each axis there is understanding of specific aspects, understanding of any one of the pituitary axes is still not complete. Here we suggest a role for networks beyond those focused on to date, to one that allows a sensing of population-level functional mass, including sensing of the cell quorum as well as secretory activity. Further analysis of the SOX2+ve cell network with the application of new methodologies allowing analysis with a detail that has not previously been possible will provide a more detailed definition of their roles and how these are coordinated. The study of pituitary cell networks and their various activities as a functional mass, as well as the interactions between both nonendocrine and endocrine cells, has important implications for health as the AP regulates a range of fundamental physiological functions and its dysregulation results in multiple diseases.

## Data Availability

Data sharing is not applicable to this article because no data sets were generated or analyzed during the present study.

## Abbreviations

ACTH: adrenocorticotropin
AP: anterior pituitary
GH: growth hormone
GHRH: growth hormone–releasing hormone
LH: luteinizing hormone
scRNAseq: single-cell RNA sequencing
SOX2+ve: SOX2-positive
